# Clinical experience and safety of Janus kinase inhibitors in giant cell arteritis: a retrospective case series from Sweden

**DOI:** 10.3389/fimmu.2023.1187584

**Published:** 2023-05-25

**Authors:** Per Eriksson, Oliver Skoglund, Cecilia Hemgren, Christopher Sjöwall

**Affiliations:** ^1^ Department of Biomedical and Clinical Sciences, Division of Inflammation and Infection/Rheumatology, Linköping University, Linköping, Sweden; ^2^ Department of Internal Medicine, Division of Rheumatology, County Hospital Ryhov, Jönköping, Sweden

**Keywords:** large vessel vasculitis, giant cell (temporal) arteritis, Janus kinase inhibitor (JAKI), baricitinib, inflammation, corticosteroids, interleukin - 6, therapy -

## Abstract

The Janus kinase (JAK)–STAT signaling pathway is relevant in both Takayasu and giant cell arteritis (GCA), and the use of JAK inhibitors (JAKi) in arthritis, psoriasis, and inflammatory bowel disease is nowadays common. Some evidence of the clinical efficacy of JAKi in GCA exists and a phase III randomized controlled trial (RCT) of upadacitinib is currently recruiting. In 2017, we started using barcitinib in a GCA patient with inadequate response to corticosteroids, and later on, we treated other 14 GCA patients with baricitinib/tofacitinib during intense follow-up. The retrospective data of these 15 individuals are here summarized. GCA was diagnosed based on the ACR criteria and/or imaging techniques combined with increased C-reactive protein (CRP) and/or erythrocyte sedimentation rate (ESR) followed by a good initial response to corticosteroids. JAKi was initiated based on inflammatory activity, with increased CRP, presumably dependent on GCA with clinical symptoms, despite unsatisfying high doses of prednisolone. The mean age at JAKi initiation was 70.1 years and the mean exposure to JAKi was 19 months. From initiation, significant reductions in CRP were seen already at 3 (*p* = 0.02) and 6 (*p* = 0.02) months. A slower decrease was observed regarding ESR at 3 (*p* = 0.12) and 6 (*p* = 0.02) months. Furthermore, the daily prednisolone doses were reduced at 3 (*p* = 0.02) and 6 (*p* = 0.004) months. No GCA relapses were observed. Two patients were affected by serious infections, but JAKi therapy was retained or reintroduced after recovery. We present encouraging observational data on JAKi in GCA in one of the hitherto largest case series with long-term follow-up. Our clinical experiences will complement the results from the awaited RCT.

## Introduction

Giant cell arteritis (GCA) and Takayasu arteritis (TAK) constitute two different types of large vessel vasculitis (1). In the elderly, GCA is often localized to the temporal arteries. However, GCA may be more widespread than generally assumed including the arteries extending from the aortic arch (2). Aortitis without the inclusion of the temporal arteries may also occur. Temporal artery biopsy (TAB) is valuable for the diagnosis of GCA and was included in the 1990 American College of Rheumatology (ACR) classification criteria (3). More recent diagnostic tools, including ultrasound (US) and computed tomography (CT) with or without the addition of positron emission tomography (PET), have improved diagnostic performance (4).

Immunomechanistic studies of GCA and TAK have revealed that dendritic cells in the arterial wall attract T cells and macrophages, and subsequently, granulomatous inflammation is formed. Several cytokines are involved in this process, for example, interleukin (IL)-6, IL-17, TNF, and interferon (IFN)-γ (5, 6). In 2017, tocilizumab (TCZ; a humanized monoclonal antibody targeting the IL-6 receptor) proved to be efficient in a randomized controlled trial (RCT) of GCA (7). Nonetheless, potentially dangerous, IL-6-blocking regimens also diminish or abolish the increase of C-reactive protein (CRP) in cases with serious infections (8–11). Patients with GCA are often old and could have multiple comorbidities combined with increased susceptibility to infection, which may hamper the enthusiasm of using IL-6-blocking therapy.

Many cytokines bind to extracellular receptors. Intracellularly, the pro-inflammatory signal of IL-6 and many other cytokines is mediated by the Janus kinase (JAK)/STAT pathway, and JAK–STAT signaling has proved important in both TAK and GCA (12–14). JAKs are subdivided into JAK1, JAK2, JAK3, and TYK2, and the effects of different cytokine receptors are mediated by pairs of these JAKs. The JAK inhibitor (JAKi) tofacitinib is considered a pan-JAKi and has shown signs of efficacy in TAK (15, 16). In 2022, Rathore et al. published a systematic review of cohort studies and case reports using JAKi in TAK or GCA (17).

The JAKi baricitinib is more selective to JAK1 and JAK2, and baricitinib has been studied in 15 GCA patients in a pilot study with promising results (18, 19). Furthermore, there are only a few case series or case reports describing the performance of baricitinib in GCA (20–22). One case of GCA treated with upadacitinib has been reported, and a randomized phase III study of upadacitinib in GCA is ongoing (SELECT-GCA) (ClinicalTrials.gov; NCT03725202) (23). In addition, one case of GCA treated with ruxolitinib has also been published (24).

By tradition, treatment of GCA starts with corticosteroids. High doses are effective, but in some patients, tapering the dose is difficult without getting a rebound of inflammation. In 2017, we started using baricitinib in a GCA patient with inadequate response to corticosteroids and where the abolishment of CRP reaction by TCZ in case of infection would have caused problems. Since then, we have treated other 14 GCA patients (who had failed on corticosteroids and/or TCZ, or where IL-6-blocking regimen with TCZ was considered inappropriate due to increased risk of, or impaired ability to diagnose, infections) with baricitinib or tofacitinib for ≥6 months, and the retrospective data of these patients are summarized herein.

## Methods

### Subjects

All subjects were diagnosed with GCA if the 1990 ACR criteria were fulfilled and/or if patients had typical US features of arteritis characterized by hypo- or medium echogenic, homogeneous, circumferential wall thickening combined with increased levels of CRP and/or erythrocyte sedimentation rate (ESR) and initially a good clinical response to corticosteroids (2). Attribution of US features to GCA was considered in each patient at follow-up, and those with other definitive or potential causes of US findings were excluded. As an alternative to US, a CT scan showing increased arterial wall thickness, or positron emission tomography CT (PET-CT) showing increased uptake of radioactive glucose in the arterial wall, was considered a sign of inflammatory activity at diagnosis.

In this retrospective study from the Swedish Rheumatology Units at the University Hospital in Linköping and the County Hospital in Jönköping, 15 consecutive GCA patients treated for at least 6 months with JAKi were included, with 14 individuals treated with baricitinib. JAKi was initiated based on inflammatory activity suspected to depend on GCA, reflected by systemic inflammation and clinical symptoms, despite unsatisfying high doses of prednisolone. For evaluation of clinical efficacy, ≥6 months of JAKi therapy was required. Patients were followed from inclusion, at 3 and 6 months, and for most of the patients, there was at least one additional time point of assessment referred to as the “last follow-up.”

### Assessments

The disease activity of GCA was evaluated by clinical investigation, including inflammatory parameters (CRP and ESR), and in two cases, imaging with US or CT was conducted. US is useful in the assessment of inflammatory activity in TAK, but the small arteries like the temporal artery are less studied (25). PET-CT was not used for the assessment of disease activity herein. All CRP levels below 5 mg/L were reported as 2.5 mg/L.

### Outcomes

The composite outcome “therapeutical benefit” was reached in subjects without suspicion of clinical activity and with the absence of new GCA symptoms (suspected to be related to GCA) and decreasing or stable CRP combined with decreasing daily prednisolone dose at 3 and 6 months post-initiation of JAKi.

In addition, 6 months after the start of JAKi, two rheumatologists independently re-evaluated if the inflammatory activity at the initiation of JAKi was attributed to GCA or if other concomitant diseases had caused the inflammation. In the latter case, the patient was withdrawn from the study.

Relapse of GCA was defined as suspicion of clinical activity based on relevant symptoms and increasing CRP (in the absence of other concomitant diseases) leading to an increase of daily prednisolone dose.

### Statistics

Data from patients exposed to JAKi were evaluated using the Wilcoxon signed-rank test to study changes over time in comparison with baseline values (SPSS software version 28.0.0.0; SPSS Inc., Chicago, IL, USA). In addition, descriptive statistics were used to display patient characteristics and laboratory values (Prism 9.3.1; GraphPad Software Inc., La Jolla, USA).

### Ethics statement

The study complied with the ethical principles of the Declaration of Helsinki. Informed consent was obtained from all patients. In Sweden, drugs are allowed to be used off-label.

## Results

### Patient characteristics

The clinical data of the 15 participants are summarized in **Table 1**. The mean age at initiation of JAKi was 70.1 (range 61−80) years, and nine (60%) were women. All patients were of Caucasian origin. None of the included patients received any other disease-modifying anti-rheumatic drugs concomitantly with JAKi.

**Table 1:** Clinical background data of the 15 study participants with giant cell arteritis (GCA).

Patient	Sex	Age at JAKi initiation (years)	Basis for GCA diagnosis	Arteritis location	DMARD at/prior JAKi initiation	Comorbidities and CVD risk factors
1	M	80	US(+), CT(+), ACR(+)	CCA, AxA, ScA	–	Angina, HT, XS
2	F	78	CT(−), TAB(−), ACR(+)	Unknown	–	HT, XS
3	F	77	US(+), TAB(−), ACR(+)	Temporal	–	HT, DM, HL, AAA, S
4	M	75	US(+), ACR(+)	Temporal	–	HT, DM
5	M	77	US(+), ACR(−)	Temporal	MTX	–
6	M	70	US(+), ACR(+)	Temporal	–	HT, DM, HL, XS
7	M	71	US(−), CT(−), PET(−), ACR(+)	Unknown	–	−
8	M	68	TAB(+), ACR(+)	Temporal	TCZ, MTX, IFX	HT
9	M	69	TAB(+), ACR(+)	Temporal	–	–
10	F	60	US(+), ACR(+)	CCA, AxA, aorta	–	–
11	F	67	US(+), ACR(−)	Brachiocephalic trunk, ScA	–	S
12	F	68	TAB(−), CT(+), ACR(+)	Aorta	TCZ	–
13	F	65	US(+), PET(+), ACR(−)	CCA, AxA, ScA, aorta	TCZ	DM, HT
14	F	65	US(+), PET(+), ACR(−)	CCA, AxA, ScA, aorta	MTX	HT
15	F	63	US(−), CT(+), TAB(−), ACR(+)	Aorta	–	XS

AAA, abdominal aortic aneurysm; ACR, 1990 ACR criteria [3]; Angina, angina pectoris; AxA, axillary artery; CCA, common carotid artery; CT, computed tomography; CVD, cardiovascular disease; DM, diabetes mellitus; DMARD, disease-modifying anti-rheumatic drugs; F, Female; HL, hyperlipidemia; HT, hypertension; IFX, infliximab; M, male; MTX, methotrexate; PET, positron emission tomography; ScA, subclavian artery; S, smoker (ongoing); TAB, temporal artery biopsy; TCZ, tocilizumab; US, ultrasound (combined with good response to steroids and exclusion of other diseases at follow-up); XS, ex-smoker.

The mean time from GCA diagnosis to initiation of JAKi was 26.9 (range 0−80) months, and the clinical manifestations at the time point of JAKi initiation (baseline) are shown in **Table 2**. The mean daily dose of baricitinib was 3.86 (range 2−4) mg, and the patients receiving tofacitinib were prescribed 10 mg daily.

**Table 2:** Individual giant cell arteritis (GCA) features and therapeutic outcomes of JAKi for the 15 study participants.

Time between GCA diagnosis and JAKi initiation (months)	GCA features at diagnosis	GCA features prior to JAKi initiation	CRP at initiation/3m/6m/last follow-up (mg/L)	ESR at initiation/3m/6m/last follow-up (mm/h)	Prednisolone at initiation/3m/6m/last follow-up (mg daily)	JAKi ongoing at last follow-up/JAKi exposure (months)	Side effects
40	CSx, PMR	HA	74/18/<5/<5	62/34/31/NA	5/5/5/5	Yes/11	None
4	HA	CSx	<5/<5/<5/17	17/54/39/NA	20/5/5/5	Yes/12	None
16	VI	CSx	23/<5/<5/7	42/13/44/45	12.5/10/7.5/5	Yes/20^a^	Bacteremia (*Enterococcus faecalis*)
8	HA, JC, CSx	HA, CSx	14/<5/<5/<5	25/8/13/12	7.5/5/2.5/0	No/29^b^	None
0	CSx	CSx	47/7/NA/7	59/19/NA/19	15/5/NA/5	Yes/6	None
7	HA, M, CSx	CSx	27/<5/<5/<5	23/6/5/6	15/5/1.25/5	No/28^b^	None
37	HA, PMR, CSx	HA, CSx	23/<5/56/<5	29/NA/34/7	15/5/5/0	Yes/24	None
80	HA, VI, CSx	M, CSx	9/<5/<5/<5	2/2/2/2	6.25/10/3.75/0	Yes/36	None
14	HA, JC, VI, CSx	HA	13/39/<5/<5	23/62/31/10	30/10/2.5/7.5	Yes/11	Infection (*Aspergillus Fumigatus*)
15	HA, CSx	CSx	27/<5/<5/<5	43/40/35/35	10/7.5/7.5/7.5	Yes/6	None
9	M, CSx	M, CSx	11/<5/<5/<5	10/11/11/14	7.5/7.5/5/0	No/26^b^	None
46	HA, M, CSx	CSx	16/<5/<5/<5	40/10/10/10	0/5/2.5/2.5	Yes/13	None
36	M, CSx	CSx	<5/<5/<5/<5	2/21/NA/33	1.25/1.25/1.25/1.25	Yes/13	None
66	PMR, CSx	M, CSx	10/<5/<5/<5	27/NA/8/9	20/10/7.5/3.75	Yes/38	None
25	HA, M, CSx	M, HA, CSx	<5/<5/<5/<5	16/10/14/12	30/10/7.5/6.25	Yes/12	None

CRP, C-reactive protein; CSx, constitutional symptoms; ESR, erythrocyte sedimentation rate; GCA, giant cell arteritis; HA, headache; JAKi, Janus kinase inhibitor; JC, jaw claudication; PMR, polymyalgia rheumatica; m, months; M, myalgia; NA, not available; VI, visual impairment.

a JAKi transiently stopped due to the side effect listed.

b JAKi ended because of sustained remission.

### Efficacy

**Figure 1:**
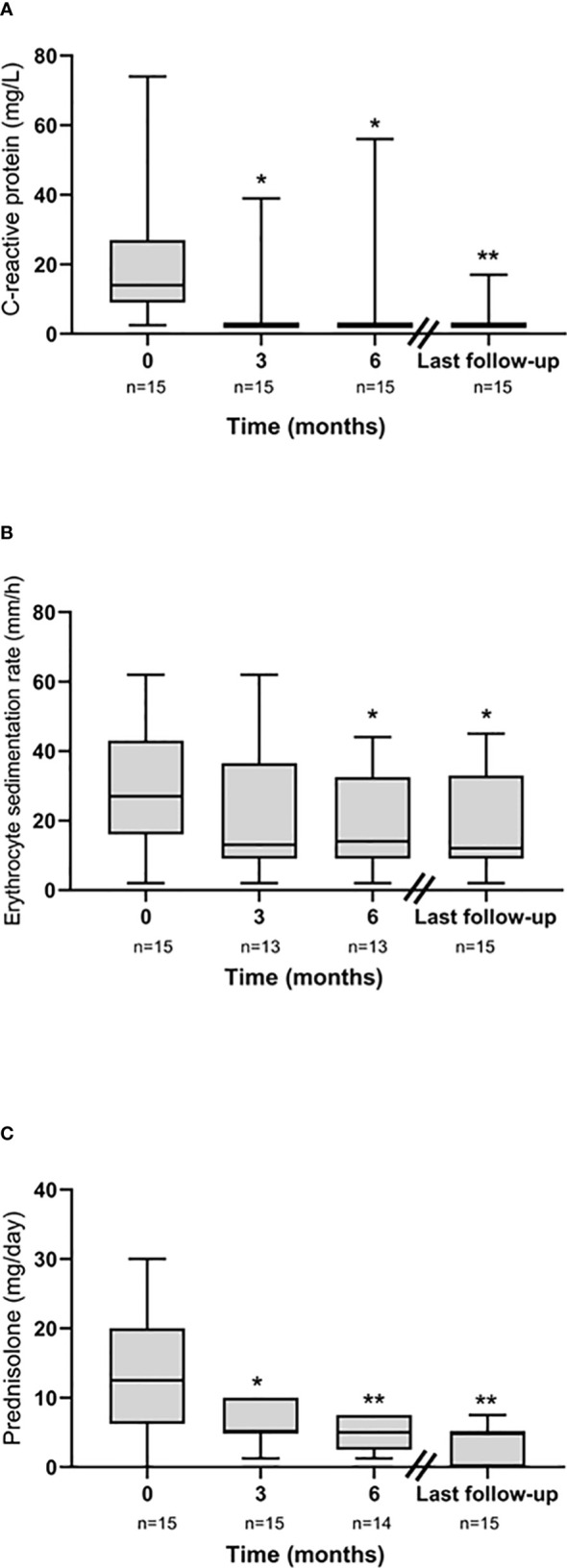
Longitudinal efficacy data from JAKi initiation (baseline) illustrated by **(A)** C-reactive protein (CRP), **(B)** erythrocyte sedimentation rate (ESR), and **(C)** daily prednisolone dose. *p < 0.05, **p < 0.01.

The mean length of exposure to JAKi was 19 (range 6−38) months. From the initiation of JAKi, significant reductions of CRP were seen at 3 (*p* = 0.02) and 6 (*p* = 0.02) months, as well as at the last available follow-up date (*p* = 0.007) (**Figure 1A**). A slower decrease was observed regarding ESR at 3 (*p* = 0.12) and 6 (*p* = 0.02) months and at the last follow-up (*p* = 0.02) (**Figure 1B**). In addition, compared with baseline, the prednisolone doses were reduced at 3 (*p* = 0.02) and 6 (*p* = 0.004) months and at the last follow-up visit (*p* = 0.002) (**Figure 1C**).

No GCA relapses were observed during the observed time. At the 3-month follow-up, 9 of 15 (60%) subjects fulfilled our definition of therapeutic benefit. This percentage increased at the 6-month follow-up when 11 of 15 (73%) individuals fulfilled the outcome of therapeutic benefit. At the last follow-up, 12 of 15 (80%) patients were still on daily treatment with JAKi. Three patients had ceased due to sustained remission after having been on the drug for more than 2 years (range 26−29 months) (**Table 2**). One of these patients experienced a GCA relapse with headache, jaw claudication, and temporal tenderness 1 month after ending JAKi. However, baricitinib 4 mg daily was reintroduced promptly in combination with a low dose of prednisolone (7.5 mg per day) to bring his symptoms under control. Consequently, he improved, and the remaining prednisolone could be tapered out over time.

### Safety

**Figure 2:**
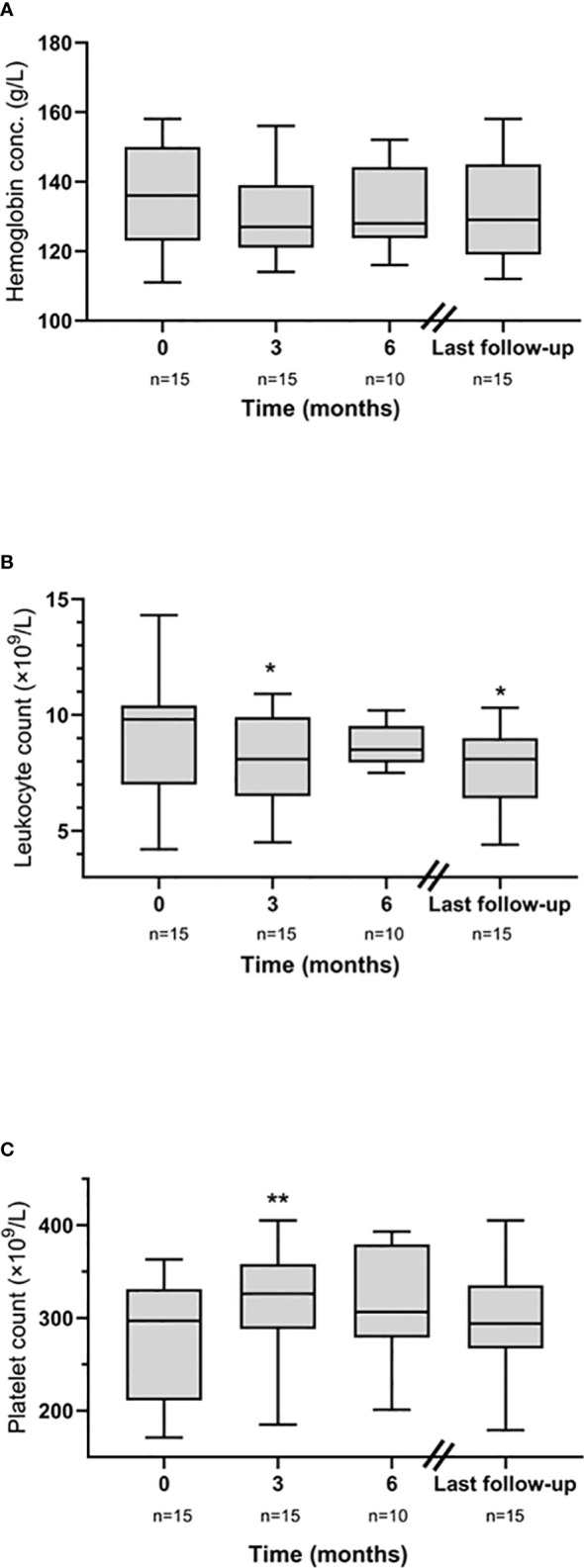
Longitudinal safety data from JAKi initiation (baseline) illustrated by **(A)** hemoglobin concentration, **(B)** leukocyte count, and **(C)** platelet count. *p <0.05, **p < 0.01.

During our study, no cases of malignancy nor gastrointestinal perforation were observed. Only minor effects were observed on blood cell counts over time (**Figures 2A−C**). Alterations of blood lipid profile, resulting in the initiation of statin therapy, were not observed in any subject. Moreover, no elevation of liver enzymes was seen, and no cases of herpes zoster were found. Nevertheless, two patients were affected by serious side effects (*Aspergillus fumigatus* infection and *Enterococcus faecalis* bacteremia, respectively). The first patient was a previously healthy 69-year-old man with GCA verified by TAB in 2020. One year later, before the initiation of baricitinib, he performed a chest CT which showed three pulmonary nodules. The nodules prompted a PET-CT, showing uptake of radioactive glucose considered a possible sign of malignancy. In February 2022 (1 month after the start of baricitinib), the size of the nodules had increased and begun to cavitate. Cultures from broncho-alveolar lavage at bronchoscopy showed *A. fumigatus*. Baricitinib was continued, and isavuconazonium was initiated, in parallel to rapidly decreasing doses of prednisolone, which led to improvement in pulmonary CT. The second patient was a 78-year-old lady with diabetes and hypertension. She had been prescribed tofacitinib for 20 months without prior episodes of infection when she was admitted to the hospital with 1 day’s duration of fevers and abdominal pain. CRP was increased (120 mg/ml) and abdominal CT showed dilated bile ducts. *Enterococcus faecalis* grew in blood cultures. The infection was successfully treated with piperacillin/tazobactam followed by amoxicillin/clavulanic acid. Tofacitinib was reintroduced to the patient as soon as she had ended the antibiotics.

### Dropouts

Aside from the 15 patients described above, three individuals were excluded from the case series as they did not prevail on JAKi for 6 months or had poor adherence to therapy.

One 85-year-old man was diagnosed with polymyalgia rheumatica in 2016. Two years later, a diagnosis of GCA was settled based on positive US findings. He also suffered from gout, recurrent infections, and chronic headaches. After 2 years, baricitinib was started in an attempt to treat headaches and systemic inflammation (increasing CRP levels). In a follow-up 2.5 months later, he had ceased baricitinib and did not notice any difference. At re-evaluation, GCA activity at the initiation of baricitinib was considered unlikely. Due to this, in combination with poor adherence to therapy, this patient was excluded from the study.

Another man was diagnosed with GCA in 2013 at the age of 74 years. Seven years later, relapse was suspected based on disturbed vision combined with a minor CRP elevation (7.5 mg/L). However, after re-evaluation 4 months later, retinal central venous occlusion per se without GCA activity was considered to explain his symptoms. Baricitinib was used for 4 months, but the patient was not included herein since attribution to GCA could not be confirmed.

Finally, a 77-year-old man with several comorbidities was diagnosed with GCA based on positive US findings and positive TAB in 2021. After 1 year on corticosteroids, baricitinib was added in order to reduce the prednisolone doses. Two months later, the patient developed shortness of breath, and a minor pulmonary embolus was observed on CT. He was treated with anticoagulation, baricitinib was ceased, and TCZ was initiated.

## Discussion

This case series, including 15 Swedish individuals above the age of 60, provides an observational real-life experience of JAKi treatment in GCA for subjects where prednisolone alone was not sufficient or when the IL-6-blocking regimen was considered inappropriate. Our study population was highly selected since the subjects had failed on corticosteroids and that IL-6-blocking regimens such as TCZ were considered inappropriate due to a lack of CRP reaction in case of an infection. Still, the majority of the patients were able to decrease their daily corticosteroids significantly after the initiation of JAKi, and the beneficial effect remained over time. Although the prednisolone doses could be tapered, CRP values were stable or decreased, and the clinical status was improved or stable for all patients.

Overall, and as indicated in **Table 2**, JAKi was well tolerated in the 15 subjects who remained on JAKi for ≥6 months. The clinical efficacy of JAKi herein appeared to be similar to that reported by Koster et al. in their 52-week proof-of-concept study using baricitinib 4 mg/day for patients with relapsing GCA (19). We observed no additional safety issues.

Assessment of inflammatory activity in GCA is not always straightforward. US as well as CT and PET-CT can support the evaluation but must be combined with laboratory and clinical data. Especially in the elderly, concomitant diseases may indeed blur the clinical picture, as illustrated by the cases with *Aspergillus* infection starting subclinically before JAKi initiation and by the dropouts where concurrent comorbidities, such as gout, confounded the evaluation of the clinical efficacy.

Aside from the two serious infections described, pulmonary embolism was observed in one patient suffering from several comorbidities after only 2 months of exposure to baricitinib. Obviously, causality between JAKi and pulmonary embolism in this patient cannot be excluded. Increased incidence of venous thrombosis and pulmonary embolism has indeed been reported in patients with rheumatoid arthritis (RA) treated with JAKi, both by the Food and Drug Administration’s adverse event reporting system and the Swedish Rheumatology Quality registers (26, 27). With regard to other adverse events and despite careful follow-up, we observed surprisingly few side effects considering that most of our patients were monitored for more than 1 year. Experience from JAKi studies on RA indicates an association with lipid profile alterations, including both high-density and low-density lipoproteins (28, 29). However, among our 15 patients, this was not observed during follow-up.

This case series must be interpreted in the context of its limitations, e.g., the absence of relevant controls. In addition, the retrospective design of the study limits the possibility to draw firm conclusions regarding efficacy. However, given the fact that the participants had failed on corticosteroids and were judged inappropriate for TCZ, relevant comparators were impossible to find. In the RCT for TCZ in the Giant Cell Arteritis trial (GiACTA), the median daily prednisolone dose in the placebo group that underwent the 26-week steroid tapering was approximately 18 mg compared with 8 mg in our study population. However, this is indeed not a perfect comparator group, especially since the same placebo group in GiACTA had a GCA duration of 365 days compared with 804 days in our study.

Safety is important but particularly challenging in the elderly population. As a reflection of this, in TCZ in GiACTA, adverse events were observed in more than 95% of the participants both in the treatment and placebo study arms (7). Another limitation of the current case series is the lack of a systematic follow-up with US or other imaging techniques. At least in arteries with a larger size than the temporal arteries, US could have given valuable information regarding the temporal development of inflammatory activity in GCA. In contrast, a major strength is the Swedish healthcare system, which is public and tax-funded and offers universal access. This significantly reduces the risk of selection bias and ensures a very high coverage of cases. Furthermore, in Sweden, existing drugs are allowed to be used off-label which is not the case for all countries.

To conclude, in line with the pilot study by Koster et al. and several independent observations, we report the encouraging efficacy data of JAKi in GCA (17, 19). The safety was comparable with prior reports in GCA. In the absence of published randomized controlled trials, clinical experience regarding efficacy and safety is important to convey to colleagues. This is one of the hitherto largest case series of JAKi in GCA with longer follow-up compared with existing reports. The clinical experiences of efficacy and safety reported by Koster et al. and us will complement the results from the awaited RCT and indicate that JAKi could be a therapeutic option in subjects with multiple comorbidities where high doses of corticosteroids and/or TCZ are considered inappropriate.

## Data availability statement

The original contributions presented in the study are included in the article/supplementary material. Further inquiries can be directed to the corresponding author.

## Ethics statement

Ethical review and approval was not required for the study on human participants in accordance with the local legislation and institutional requirements. The patients/participants provided their written informed consent to participate in this study. Written informed consent was obtained from the individual(s) for the publication of any potentially identifiable images or data included in this article.

## Author contributions

PE: original idea, patient characterization, manuscript writing, and supervision. OS: patient characterization, manuscript writing, and statistics. CH: patient characterization and manuscript writing. CS: patient characterization, manuscript writing, and supervision. All authors reviewed the manuscript and approved the final version.
